# Why Public Health Is Not About Public Goods

**DOI:** 10.1093/phe/phaf023

**Published:** 2025-11-27

**Authors:** Lovro Savić

**Affiliations:** Ethox Centre, Nuffield Department of Population Health, University of Oxford, Oxford, UK

## Abstract

According to the *Public Goods Account*, proposed by Jonny Anomaly, public health activities should only be concerned with the provision of *health-related* public goods. In this paper, I argue that the *Public Goods Account* cannot serve as an adequate account of public health activity. The main reason is that its central concept, that of health-related public goods, is itself implausible. I offer two potential understandings of health-related public goods and argue that, on both understandings, the provision of health-related public goods is neither necessary nor sufficient for a public health activity. First, on both understandings, there are plausible examples of public health activities that do not produce health-related public goods. And second, there are examples of non-public health activities that produce health-related public goods.

## Introduction

Like other related ‘health’ concepts,^1^ how we understand ‘public health activity’ and how we define it carries many important consequences. Perhaps most obviously, how we define public health is in an important way inextricably linked to the distribution of professional duties (See for example: Tengland, 2015), scarce resources, aid and research funding (Giubilini, 2025), that typically have crucial implications for the health of the individuals and populations. More importantly, however, it also carries normative relevance. For example, any adequate account of public health *ethics* will unavoidably rest on how we define public health activity (Dawson, 2011). This idea seems to rest, at least in part, on the observation that the aims, nature and methods of public health activities are in some substantive sense different from those of clinical medicine (Dawson, 2011). To the extent that this is true, then applying current theories and principles of medical ethics might be inappropriate for answering normative questions in the domain of public health. That is, if our task is to examine the moral permissibility of public health activities, we must be able to identify properties that allow us to class them as public health activities rather than something else. Without a proper definition, we run the risk of being unable to show what *exactly* makes carrying out public health activities morally (im)permissible or whether or not our arguments are actually successful in morally justifying something other than public health activities.^2^ In other words, ‘it is vitally important to be clear about what we mean by public health before we begin to explore public health ethics’ (Dawson, 2011, p. 5).

According to Jonny Anomaly (2011), public health activities should only be concerned with the provision of health-related public goods. Call this *Public Goods Account*. Anomaly insists that by focusing on the concept of health-related public goods, *Public Goods Account* offers several important benefits. First, it appears to be an account of public health activity that is both clear and simple. Second, it captures the original and traditional mission of public health by distinguishing public health activities from other related, but nevertheless distinctive health care activities. And third, it is compatible with a wide range of normative positions and, therefore, avoids the issue of political disagreement (Anomaly, 2011).

These three benefits are important. A clear and simple account has a good chance of being understood by the general public. As such, it does not stand in the way of public participation or compliance with public health policies and regulations. *Public Goods Account* can also successfully avoid distributive dilemmas between different areas of healthcare. It can clearly single out those activities that produce *public*, rather than *private* health-related goods, such as individual medical care. Lastly, since it is compatible with a wide range of moral and political theories, it avoids controversial normative judgments about the moral permissibility of specific public health activities and interventions (Anomaly, 2011).

In this paper, I argue that *Public Goods Account* cannot serve as an adequate account of public health. The main reason is that its central concept, that of health-related public goods, is itself implausible. According to Anomaly, there are two ways to understand health-related public goods. On one view, to say that public goods are health-related is to say that they are ‘associated with medicine’ (Anomaly, 2011, p. 251). I will call this view, *Public Goods Account*_Medicine_. According to another, alternative view, something is a health-related public good insofar as the benefits it produces have a positive population-level effect on peoples’ health (Anomaly, 2011, 2021). I will refer to this, second view as *Public Good Account*_Outcome_.

In this paper, I argue that, unfortunately, both views are inadequate. First, I provide an overview of the concept of public goods and two understandings of specifically *health-related* public goods endorsed by Anomaly. In the second part, I offer several objections to *Public Goods Account*_Medicine_. I recount and offer a further defence of Bernstein and Randall’s (2020) and Dees’ (2018) claim that, once understood as those public goods that are ‘associated with medicine’, health-related public goods are *not necessary* for a public health activity. That is, there are paradigmatic examples of public health activities that produce public goods that are *not* associated with medicine. I also advance a novel claim that, understood in the same way, health-related public goods are *not sufficient* for a public health activity, either. There are plausibly some activities that produce public goods that are associated with medicine, but we have sufficiently strong reasons not to class these activities as genuine public health activities. Therefore, insofar as it is understood as *Public Goods Account*_Medicine_, *Public Goods Account* is implausible.

In the third part, I argue that the alternative, *Public Good Account*_Outcome_ is equally problematic. Understood as those public goods, *the benefits of which have positive population-level effects on peoples’ health,* health-related public goods are *neither necessary nor sufficient* for a public health activity. To show this, I argue that there are genuine public health activities that produce positive population-level effects on peoples’ health, but these outcomes are neither *pure* public goods, nor are they public goods *tout court*. Secondly, I argue that there are a number of legitimate public health activities that produce outcomes that indeed constitute public goods, but these public goods are *not* health-related in the required sense because they either do not produce positive population-level effects on people’s health, or when they do, the resulting effects are too imperceptible and insubstantial to warrant the status of a public health activity. This includes conceivable public health activities that are concerned with allergies and rare diseases. Therefore, understood as *Public Good Account*_Outcome_, *Public Good Account* is also implausible.

If the arguments presented are plausible, then we have a sufficiently compelling reason to doubt that *Public Good Account* can serve as an adequate account of public health activity. However, while the lessons to be drawn from these arguments are unavoidably sceptical, they are also in some, important sense *ameliorative*. That is, while it is certainly plausible to assume that virtually any account of public health activity will face some problematic cases and counter-intuitive implications, this should not discourage us from the idea that we can indeed make significant and substantive progress in defining public health activity. Therefore, the lessons generated by the analysis of *Public Good Account* put us in a considerably better position for coming up with a positive and more adequate definition of public health activity which either strives to keep the number of problematic cases at minimum or which, perhaps, solves the most problematic and difficult ones.

## Public Goods and Health-related Public Goods

Let us start with the concept of public *goods*. On the most general construal, public goods refer to any physical commodity or service that produces perceived benefits which cannot be denied to others (Klosko, 1987). In other words, public goods produce benefits that are *non*-excludable and *non*-rivalrous in consumption (Woodward and Smith, 2003). In the context of public health, a standard example of a public good is herd immunity. Once achieved, herd immunity offers a benefit that is both *non*-excludable and *non*-rivalrous in the sense that the quality and quantity of protection from an infectious disease is available to and equal for everyone. First, this is because it is impossible to exclude someone from benefiting from the protection conferred by herd immunity. And second, the extent to which some people benefit from the protection against an infectious disease does not reduce or restrict the extent to which others benefit from it (Giubilini *et al*., 2018).

Importantly, however, on *Public Goods Account*, public health activities do not provide just *any* kind of public goods. On Anomaly’s view, public health activities are concerned with the provision of specifically *health-related* public goods. The main question here is what makes a public good a specifically health-related public good?

Anomaly answers this question in the following way:

The thesis of this essay is that public health should be concerned with the provision of public goods *associated with medicine*. More speciﬁcally, public health should attempt to promote health and prevent disease among populations of people for whom health outcomes exhibit the two characteristic features of public goods: non-excludability and non-rivalry (Anomaly, 2011, p. 251).

According to Bernstein and Randal (2020), however, Anomaly’s answer has been taken to involve two sufficiently different views. According to what I will refer to as *Public Goods Account*_Medicine_, to say that a public good is health-related is to say that the public good is *associated with medicine*. According to the second and considerably broader (Bernstein and Randall, 2020) *Public Goods Account*_Outcome_, something is a health-related public good insofar as the benefit it produces has positive population-level effects on peoples’ health (Anomaly, 2011, 2021). Indeed, as it will become obvious throughout this paper, *Public Goods Account*_Medicine_ and *Public Goods Account*_Outcome_ are neither conceptually equivalent nor is the latter view is merely a specification of the former since there are objections that apply to one view rather than the other.^3^

## Public Goods that are Associated With Medicine are Not Necessary for a Public Health Activity

Let us first start with the objection against *Public Goods Account*_Medicine_, which states that being *associated with medicine* is *not necessary* for a public health activity. This objection is not completely new. It rests, at least in part, on the conjunction of claims made by Bernstein and Randall (2020) and Dees (2018). Dees, for example, correctly observes that ‘the most important advances in public health in the past 200 years are the result of clean water and proper sanitation’ (Cutler and Miller, 2005; *in* Dees, 2018, p. 22). More importantly, Bernstein and Randall similarly point out that sanitation and water treatment are examples of public health activities that ‘are not […] related to medicine in any discernible sense’ (2020, p. 227).

If true, these claims put *Public Goods Account*_Medicine_ in ‘double jeopardy’. First, excluding sanitation and water treatment would make *Public Goods Account*_Medicine_ too narrow. After all, many of us would have a strong inclination to agree with Dees and class sanitation and water treatment as instances of public health activities. And second, these activities have been traditionally praised as the classics of preventative public health (Bayer *et al*., 2007). Therefore, excluding these activities would defeat Anomaly’s explicit goal of reclaiming the traditional conception of public health (Bernstein and Randall, 2020).

However, while powerful, these objections are not immediately obvious. The success of these claims is, to a large extent, predicated on our understanding of the expression ‘associated with medicine’. On the simplest understanding, we could take *Public Goods Account*_Medicine_ to mean that public goods are associated with medicine or medical intervention *itself,* that is, with healing diseases and curing the sick in the sense of ‘diagnosis and treatment of individual patients in institutional health care contexts’ (Holland, 2019, p. 1). But Anomaly cannot accept this claim. The first reason is that this understanding of *Public Goods Account*_Medicine_ would make the concept of public health activity indistinguishable from the concept of individual medical care. Second, and relatedly, insofar as the *Public Goods Account* is taken to have the advantage over competing accounts of public health in terms of ‘establishing a relatively *clear and distinctive mission* of public health’ (Anomaly, 2011, p. 251, *my emphasis*), endorsing this understanding would make *Public Goods Account*_Medicine_ fail on its own terms.

Another possibility, however, is to understand ‘associated with medicine’ without any reference to health, disease, cure or treatment. In doing so, we would be endorsing the view of the sort put forward by Lawrie Reznek. According to Reznek, medicine and medical interventions can be defined purely ‘enumeratively’, in terms of ‘pharmacological and surgical interventions’ (Reznek, 2022, p. 163; See also: Cooper, 2002). Endorsing Reznek’s understanding, however, is not entirely suitable for *Public Goods Account*_Medicine_. While many public health activities, such as vaccinations, involve pharmacological interventions, there are very few—if any—public health activities that involve surgical interventions. To avoid this problem, we could broaden our understanding and take *Public Goods Account*_Medicine_ to refer to those public goods that are provided by *means*, *tools* and *methods* standardly employed in medical practice. Many would, I believe, have a strong inclination to accept this idea. After all, we rely on the expertise of *medical* practitioners in our fight against pandemics. When we come forward to boost our immune system against an infectious disease, they poke our arms with a *medical* instrument (i.e. syringes), inject a *medical* product (i.e. vaccine) and so on.

However, even on this understanding, *Public Goods Account*_Medicine_ remains vulnerable to objections of the sort raised by Dees (2018) and Bernstein and Randall (2020). First, it cannot account for obvious public health activities that produce public goods using obviously *non*-medical means. Second, it cannot explain examples of public health activities that produce public goods using means, tools and methods that are not *exclusively* or *obviously* medical.

### Public Health Activities that Use Obviously Non-medical Means

In simplest terms, the first objection against *Public Goods Account*_Medicine_ states that there are public health activities, the outcomes of which take the form of public goods that are produced by obviously *non*-medical means. Two examples are removing asbestos and galvanized pipes from residential or occupational buildings and areas. Many of us would be strongly inclined to class these as examples of public health activities. But *Public Goods Account*_Medicine_ can only *partially* explain this. First, on this account, in removing asbestos and replacing galvanized pipes, these public health activities would produce public goods. This is because, insofar as these activities are successful, they minimise risks in the physical environment and contribute to the health of everyone living in that environment (Verweij, 2007). In other words, the benefits of protection from hazardous materials and substances would be available to all in the sense that no one can be restricted or excluded from enjoying the protection. Therefore, the outcomes of removing asbestos or galvanized pipes constitute public goods.

However, while it can explain what makes the outcomes of removing asbestos or galvanized pipes an instance of public goods, *Public Goods Account*_Medicine_ cannot explain what makes these outcomes specifically *health-related* public goods. We can illustrate this objection in the following way. When we discover a hazardous deposit of asbestos, we rely on construction workers and their expertise to remove it, rather than on medical nurses and doctors. We remove it using *non*-medical tools and methods such as the demolition of walls and floors, and the use of cranes, hammers and lorries, rather than, say, X-ray machines, syringes, or vaccines. But on *Public Goods Account*_Medicine_, these interventions would not count as public health activities. This, however, is something that virtually no one would be ready to accept. Therefore, once understood as those public goods that are ‘associated with medicine’, *health-related* public goods are *not necessary* for a public health activity.

### Public Health Activities that Use Medical Means that are not Exclusively Medical

The second objection against *Public Goods Account*_Medicine_ states that once understood as those public goods that are ‘associated with medicine’, *health-related* public goods are not necessary for a public health activity because there are public health activities the outcomes of which take the form of public goods produced by using means, tools and methods that are *not exclusively* or *obviously* medical. An apparent example involves wearing face coverings in a pandemic. Again, following Verweij’s (2007) claim from above, we can take policies mandating the wearing of face coverings to belong to a category of public health activities that reduce risk factors that are part of the physical or the social environment of a larger public. If successful, wearing face coverings makes the physical and social environment such that, in a place where one is likely to catch a virus, this likelihood is greatly reduced or even completely eliminated. And since everyone benefits from such an environment (Verweij, 2007), the outcome of wearing face coverings would, plausibly, constitute a public good.

However, while many of us would class wearing face coverings as an instance of a public health activity, it is far from evident that face coverings are *obviously* or even *exclusively* medical tools. At first sight, this objection might seem odd. What seems to elicit this reaction is just the fact that we standardly associate face coverings with medical practitioners and medicine in general. After all, it seems difficult to think of a doctor without the image of a person in scrubs, holding a stethoscope and wearing a face mask coming to our minds.

We might, perhaps, try to explain the wide-spread inclination that face coverings are medical tools with reference to the idea that they were designed with the specific medical purpose of preventing infections. In accepting this idea, we are also endorsing the claim that the purpose of an object can be determined on the *description of properties of the object itself* together with the reasonable assumptions *about the purposes of the people by whom they were designed* (Fulford, 1989).

The first, swift response here is that there is simply nothing about the piece of cloth that we put over our mouths and noses that makes it a medical tool. Second, *even if* we can make reasonable assumptions about the purposes for which medical masks have been made, this still does not explain all types of face coverings used during pandemics. For example, the most reasonable source of confirmation that something is a medical tool is that the object in question belongs to a class of objects that are *currently defined* as ‘medical devices’. According to one definition, for example,

a medical device can be any instrument, apparatus, implement, machine, appliance, implant, reagent for *in vitro* use, software, material, or other similar or related article, *intended by the manufacturer to be used*, alone or in combination *for a medical purpose* (WHO, n.d., *my emphasis*).

However, many face coverings worn during a pandemic are not of the sort intended by the manufacturer to be used for medical purposes. While some people might wear simple ‘self-made’ cloth face coverings, ‘neck gaiters’ or ‘balaclavas’, others might wear, say, ‘N95 respirators’. In many cases, for example, balaclavas or neck gaiters are designed and intended by manufacturers to be used for, say, military or sports-related purposes, or as a fashion trend. Even in the case of N95 respirators, it is simply not true that *all* N95 respirators are ‘produced with an intention’ to serve a medical purpose. In fact, N95 respirators are an example of ‘personal protective equipment that is used to protect the wearer from particles or from liquid contaminating the face’. In other words, while ‘*some* N95 respirators are intended for use in a healthcare setting’, ‘*most* N95 respirators are manufactured for use in construction and other industrial-type jobs that expose workers to dust and small particles’ (U.S. Food and Drug Administration, 2023, *my emphasis*). And so, since balaclavas or N95 respirators are intended by the manufacturer to be used for *non*-medical purposes, this generates sufficiently compelling reasons to justify the claim that—at least in some instances—face coverings worn during a pandemic are not obviously or exclusively medical tools. This, in turn, generates a sufficiently compelling reason to believe that the production of public goods that are associated with medicine is *not a necessary* condition for a public health activity. And if this is correct, then *Public Goods Account*_Medicine_ is inadequate.

## Public Goods that are Associated With Medicine are Not Sufficient for a Public Health Activity

### Collective Moral Bioenhancement

I now turn to the objection that *Public Goods Account*_Medicine_ is inadequate because, once understood as those public goods that are ‘associated with medicine’, *health-related* public goods are *not sufficient* for a public health activity either. This objection rests on the observation that there are plausibly some activities that produce public goods with medical means, tools, or methods, but we have sufficiently compelling reasons not to class these activities as genuine public health activities.

To illustrate, consider the example of a *Collective Moral Bioenhancement* programme (Crutchfield, 2019, 2021). One potential example is water supply fortification with *oxytocin-*like chemical compounds, a class of *drugs and medicines* that we have good reasons to believe promote pro-social and moral behaviour and improve social cognition and empathy (See, for example: Conan, 2020). Suppose that, unlike the current iterations, the modified version allows us to predictably and safely lessen hostility and biases against members of other social groups. And so, should such an intervention become technologically feasible, it might soon be possible to collectively enhance moral attitudes, motivations, or behaviour of entire populations so that harms resulting from violence or unjust discrimination (Douglas, 2008; Conan, 2020) are minimized or completely eliminated. And since *oxytocin* belongs to classes of drugs and medicines, and since unjust discrimination- and violence-free social environment would be *available to everyone* in the sense that no one would be restricted or excluded from enjoying the benefits of being protected from harms associated with violence or unjust discrimination, *Collective Moral Bioenhancement* would, strictly speaking, produce a health-related public good and would, on *Public Goods Account*_Medicine_, count as a public health activity. However, in proposing a *traditionally narrow* conception of public health, Anomaly *himself* simply cannot accept this conclusion. According to him, activities that aim at the social determinants of health, promote peace, or address racism and violence belong to social welfare interventions, and *should not* be considered part of public health (Anomaly, 2011).

## Public Goods that Have Positive Population-level Effects on People’s Health are Not Necessary for Public Health Activities

According to the alternative, *Public Goods Account*_Outcome_, to say that a public good is health-related is to say that the benefits it produces *have positive population-level effects on peoples’ health*. In Anomaly’s words,

… public health should attempt to promote health and prevent disease among populations of people for whom health outcomes exhibit the two features characteristic of public goods: non-excludability and non-rivalry (Anomaly, 2011, p. 251).

One obvious advantage of this view is that it is not sensitive to the way public goods are produced. We can produce a public good using medical tools such as syringes and pharmacological agents, or we can produce it using hammers, drills and computers. And since the way we produce public goods does not matter, *Public Goods Account*_Outcome_ can account for public health activities such as wearing masks or removing asbestos and galvanized pipes.

The plausibility of *Public Goods Account*_Outcome_, however, will depend on our understanding of ‘population-level health outcomes’. On Anomaly’s view, populations are simply groups of individuals­­—there is no separate, ‘meta-person’ or entity that can be healthy or diseased, benefited or harmed (Anomaly, 2011). So, in accepting this view, Anomaly seems to endorse a version of Verweij and Dawson’s idea that public health activities are the type of activities that are expected to make a population-level difference—that they should affect the health of *many*. On this understanding, public health activities involve producing positive population-level effects on peoples’ health in the sense of improving (or maintaining) the aggregate health of all members of the population (Verweij and Dawson, 2007).

However, even on this interpretation, health-related public goods are not necessary for a public health activity. The main reason for this is that there are plausibly some public health activities the outcomes of which have positive population level effects on peoples’ health, but these outcomes are either *im*pure public goods or are not public goods *tout court*.

### Public Goods Tout Court

The most obvious example of a public health activity, the outcomes of which take the form of improved aggregate health of the population, but which do not constitute public goods *tout court*, is vaccination against tetanus and other non-infectious diseases. In vaccinating people against *tetanus*, fewer people get ill and we maintain or improve the aggregate health of the population. On this view, then, the vaccination programme against tetanus produces positive population-level effects on peoples’ health. But vaccination against tetanus produces *private goods* rather than *public goods*. This is because, first, tetanus is not contagious, and vaccines do not produce herd immunity (public good) (Dees, 2018). And second, *Clostridium Tetani*, the bacteria that cause tetanus, are common in the environment and vaccination does not have any effect on their presence in the environment (Dawson, 2007). And since I cannot infect others with tetanus, and since my vaccination does not minimize risks of infection from the environment, *no one benefits* from the fact that any particular individual is vaccinated (Dawson, 2007).

### Impure Public Goods

The second, related objection against *Public Goods Account*_Outcome_ maintains that there are public health activities that improve the *aggregate* health of the population, but their outcomes do not constitute *pure* public goods. *Pure* public goods are those public goods that are simultaneously both *fully* non-excludable and *fully* non-rivalrous and, therefore, *available to all* (Woodward and Smith, 2003), in the sense that for each person who is a position to benefit from the good, this is, *at least in principle*, possible (Kallhoff, 2014). *Im*pure public goods, on the other hand, are those public goods that are either not fully non-excludable or not fully non-rivalrous, or both (Woodward and Smith, 2003).

An example of a public health activity that produces *im*pure public goods involves tobacco control policies, such as legal age limits. In most countries where legal age limits are enforced, individuals under 18 or 21 years of age are prohibited from purchasing tobacco products. Legal age limits, once instituted, can plausibly be said to modify the social environment in a way that it is easier for adolescent-to-be-smokers to refrain from starting smoking and for adolescents who currently smoke to stop smoking (see: Verweij, 2007). Insofar as these activities are successful, legal age limits produce positive population-level health outcomes in the sense that they maintain an aggregate level of health in a population that would otherwise be diminished due to the presence of smoking-related diseases.

However, at the core of this objection is that these outcomes do not constitute *pure* public goods. Even if fully successful, legal age limits do not produce benefits that are *fully non*-excludable and fully *non*-rivalrous. This is because they offer the benefits of protection from smoking-related harms that can be enjoyed *only* by a certain *group* that is defined by age, namely, minors and adolescents. For example, access to the benefits of protection from smoking-related harms available to minors and adolescents remains *permanently closed* to me as a 35-year-old man. Therefore, while legal age limits indeed produce positive population-level health outcomes in the sense that they maintain an aggregate level of health in a population, these outcomes constitute *im*pure, permanently closed, *group* public goods.

If these arguments are plausible, they seem to generate sufficiently compelling reasons for the conclusion that, understood as those public goods the benefits of which have positive population-level effects on peoples’ health, health-related public goods are not *necessary* for a public health activity.

## Public Goods that Have Positive Population-level Effects on People's Health are Not Sufficient for Public Health Activities

The second objection against *Public Goods Account*_Outcome_ is that, understood as those public goods that have a positive population-level effect on peoples’ health, health-related public goods are not *sufficient* for a public health activity either. In other words, there plausibly are such public health activities that produce public goods, but these public goods are not health-related in the sense that they *do not* produce positive population-level effects on people’s health, or if they do, these effects are insubstantial and imperceptible.

### Anti-allergy Spray

Consider, first, a conceivable public health activity that is aimed at preventing symptoms of hay fever, a common allergic reaction to allergenic pollen grains produced by flowers, grasses and trees (McInnes, 2019). This activity might consist in, say, introducing powerful anti-allergy aerosol sprays in areas known to have high concentrations of pollen. When applied, anti-allergy aerosol sprays dispense a mist of liquid particles containing antihistamines that, once inhaled, neutralize or significantly relieve unpleasant symptoms of pollen allergy. Call this the *anti-allergy spray*.

*The anti-allergy spray* modifies the environment so that the impact of allergenic pollen is suppressed or completely alleviated. And this environment would be ‘open to all’ in the sense that it would be possible, at least in principle, for every person to benefit from the good. Furthermore, the fact that some people enjoy the protection from the unpleasant symptoms of hay fever does not reduce—at least in principle—the quality or quantity of protection accessible to everyone else. In a very robust sense, then, the outcome of *the anti-allergy spray* would constitute a public good.

However, the *anti-allergy spray* is problematic for *Public Goods Account*_Outcome_ in that, according to Anomaly, positive population-level health outcomes should result from either health promotion or disease prevention (Anomaly, 2011). But the *anti-allergy spray* does not promote health or prevent disease.

### The Nature of Allergies

The main reason in favour of this claim has to do with the nature of allergies. On a widely endorsed naturalistic understanding, health is simply equated with normal species functioning (Boorse, 1997; Varga, 2011). In other words, an organism is healthy ‘if all its *functional* parts are able to function in a statistically normal way, given its species, sex, and age’ (Werkhoven, 2020, p. 145, *my emphasis*; Boorse, 1977). For example, the function of the heart’s sinus node is the production of electrical signals that cause the contraction of the myocardium, which causes the heart to pump blood and supply the body with oxygen, which in turn, contributes to keeping the organism alive (Boorse, 1976; Cooper, 2005). Diseases, on the other hand, are defined as interference with natural functions (Boorse, 1976). And so, a heart attack is a disease precisely because it intervenes with and prevents the heart from performing its function—pumping blood, and keeping the organism alive (Boorse, 1976; Cooper, 2005).

However, in accepting ‘disease-as-dysfunction’ account, we would also have strong reasons to reject the claim that allergies are diseases. In simple terms, upon exposure to foreign substances—allergens—the reaction of allergic people standardly involves swollen eyes, sneezing, skin irritation, itchy throats and so on. These reactions are a result of histamines—chemicals produced by the mast cells of our immune system (Ananth, 2008) However, according to one potential explanation, allergies are designed to be the ‘last line of defence’ against toxins when the primary antitoxin defence mechanism fails (Profet, 1991; Ananth, 2008). In other words, the function of mast cells *is* the production of histamines, that is, mast cells are *doing precisely what they are supposed to do* in the presence of allergens (Ananth, 2008). But if this is a correct characterisation of allergies, allergic reactions are, in fact, a sign that the immune system is functioning *properly*. Therefore, since they do not involve any dysfunction, allergies are not diseases. And since they are not diseases, the *anti-allergy spray* does not produce positive population-level health outcomes in terms of disease prevention. On *Public Goods Account*_Outcome_, then, the *anti-allergy spray* would not constitute a public health activity.

On another, supporting view, it could be said that allergies are not diseases because they belong to the class of vestigial systems (Ananth, 2008). Vestigial systems, such as the appendix, the tailbone and goosebumps, are those components of an organism that presently do not have any function or purpose (Werth, 2014). For those who accept the idea that allergies are, in fact, vestigial systems, allergies are much like the appendix in the sense that they can, in some cases, cause physiological problems, but have no present function (Ananth, 2008). On this view, then, allergies are not dysfunctions because they do not serve any function to begin with. And since allergies do not serve any function or dysfunction, they do not constitute a disease.

If the arguments examined in this section are plausible, then we have good reasons to accept that the *anti-allergy spray* does not produce positive population-level health in terms of either health promotion or disease prevention. And if this is correct, then the *anti-allergy spray* supports the claim that in accepting *Public Goods Account*_Outcome_, we also have sufficiently strong reasons to accept that health-related public goods *are not sufficient* for public health activities.

#### Anti-allergy spray and the requirement of safety

As a way of response, Anomaly might claim that we have reasons to doubt that the *anti-allergy spray* constitutes a genuine public health activity because it is not at all evident that such an activity would be safe.^4^ This worry, however, can be satisfactorily answered by appealing to the fact that there is nothing conceptually inconsistent or unsound about the idea of *unsafe or harmful* public health activities. Not only do public health activities often fail at solving a specific public health problem (Wilson, 2021) but they also frequently increase the very adverse outcomes they seek to prevent (Bavli *et al*., 2020), affect other health outcomes (Bavli *et al*., 2020), and often have unintended harmful effects (Lorenc and Oliver, 2014). For example, there is now an increasing amount of studies that lend support for the claim that recent COVID-19 pandemic responses, such as nation-wide lockdowns, had a number of harmful effects in the sense of negatively impacting a range of pre-existing health conditions (Brown *et al*., 2021), compromising people’s immune systems (Schippers, 2020), and increasing prevalence of anxiety and depression (Dettmann *et al*., 2022). Yet, virtually everyone would have a strong inclination to classify the nation-wide lockdowns as public health activities *despite* their harmful effects.

Perhaps the most obvious reason in favour of this inclination rests on the observation of how the requirement of safety relates to, say, instances of individual medical interventions. Many individual medical interventions often fall short of complete safety and carry some degree of harm. However, the fact that, say, neurosurgeries or chemotherapies are often unsafe and that they carry a significant risk of harmful side-effects does not entail that these interventions should be stripped of the status of medical intervention. But if we are compelled to accept that the lack of safety should not have an effect on the status of chemotherapies and neurosurgeries as individual medical care interventions, then we are also forced to endorse the claim that the fact that public health activities are insufficiently safe does not make a compelling case that these activities should be, for that very reason, stripped of *their* ‘public health’ status, either.

#### Anti-allergy spray and broader accounts of disease

The second potential objection maintains that the success of *the anti-allergy spray* is predicated on overly narrow, *Biostatistical Theory of Health*. That is, while allergies cannot be classed as diseases on the *Biostatistical Theory of Health*, they can be classed as such according to other, considerably broader theories of health.^5^ According to one potential version of this response, Anomaly could endorse Huber’s account of health (Huber *et al*., 2011). On Huber’s account, ‘health’ refers to the ability to adapt and to self-manage in the domains of one’s physical, mental and social health (Huber *et al*., 2011). And so, even if we grant the assumption that hay fever does not constitute a disease on *Biostatistical Theory of Health*, it does constitute a disease on Huber’s definition of health, since the unpleasant signs and symptoms of pollen allergy can plausibly be taken to diminish one’s capacity and ability to adapt and self-manage in the domain of, say, mental and physical health.

However, endorsing Huber’s account of health leads to a serious implication that Anomaly cannot accept. The strength of this claim rests, at least in part, on what we understand by the term ‘social health’. According to one understanding, social health refers to ‘adequate quantity and quality of relationships in a particular context to meet an individual’s need for meaningful human connection’ (Doyle and Link, 2024, p. 626). However, in accepting this understanding of social health, we would also be forced to seriously consider the idea that activities that attempt to promote people’s ability to adapt to, self-manage or prevent loneliness^6^ (including both sexual and romantic loneliness) should be classed as genuine public health activities. This, however, would strike most people as unacceptably counterintuitive, if anything, then for the simple reason that activities that prevent loneliness are more reasonably classed as instances of social (or, perhaps, private) *welfare* interventions. In addition, just like in the case of *Collective Moral Bioenhancement*, in proposing a *traditionally narrow* conception of public health, endorsing Huber’s account of health is something that Anomaly *himself* cannot accept.

Perhaps another way to avoid the objection raised by *the anti-allergy spray* might include endorsing Venkatapuram theory of health. According to Venkatapuram, health is ‘a person’s capability to achieve, exercise or express (‘achieve’) a cluster of basic and inter-related capabilities and functionings’ (Venkatapuram, 2011, p. 31; See also: Tengland, 2016, p. 12), such as capabilities to: (i) live a normal life span, (ii) be fit and stay in good health, (iii) have and experience bodily integrity, including freedom to move around and make reproductive choices, (iv) use her senses and imagination, and be able to think, (v) experience emotions and have emotional attachments, (vi) exercise (her) practical reasoning in order to form a conception of the good, and critically reflect on different goods, (vii) establish affiliations, that is, live with others and have a social basis for self-respect, (viii) live with, and express concern for, other species, (ix) play, laugh and enjoy recreational activities, and (x) participate effectively in political choices, and control the social and physical environment, including to hold property and seek employment (Nussbaum, 2013; Tengland, 2016). In endorsing Venkatapuram’s theory of health, *Public Goods Account*_Outcome_ can account for the *anti-allergy spray* as an instance of a genuine public health activity because some of the capabilities might be negatively affected by the pollen allergy.

However, it is easy to notice that endorsing Venkatapuram's theory of health is equally problematic. The first, most straightforward reason is that it would entail that public health activities are concerned with the provision of those public goods that promote people’s capabilities to establish affiliations, and life and relationships with others. However, this would also make *Public Goods Account*_Outcome_ too broad an account that Anomaly simply cannot accept. The second reason rests on the idea that it can be doubted whether the proposed capabilities are really ‘objective goods’, if the ones included in the list are indeed the most valuable ones (Tengland, 2016), and whether there are, perhaps, *other* capabilities that we might be justified in including on the list. This, however, would defeat Anomaly’s explicit goal of coming up with an account of public health that is compatible with a wide range of normative positions and that avoids disagreements and controversial normative judgments about the moral permissibility of specific public health activities and interventions (Anomaly, 2011).

### Rare Diseases

The final objection against *Public Goods Account*_Outcome_ maintains that *even if* we assume that the concept of disease is best understood and explained with reference to an account of health other than the *Biostatistical Theory of Health*, and *even if* we assume that allergies can be classed as diseases, this would still not save *Public Goods Account*_Outcome_. The reason is that there are plausible instances of public health activities that produce population-level effects on peoples’ health in terms of preventing diseases, but these effects are too *imperceptible* to grant the activity the status of a public health activity that produces health-related public goods.

One example of such activities includes screening programmes for some types of genetic disorders. *In general*, screening programmes are commonly considered as a major branch of public health (Juth and Munthe, 2011; Holland, 2015) and are typically carried out as a way of selecting those at risk of disease or disorder for possible further treatment (Juth and Munthe, 2011). Insofar as there is acceptable treatment associated with the programme, the goal of screening programmes is to reduce morbidity or mortality in the population (Juth and Munthe, 2011). Consider, e.g. a newborn screening programme for PKU. PKU is one of the very few serious monogenetic diseases that can be easily and effectively prevented. Once detected, this can be done with continuous, life-long dietary modification of restricting the intake of phenylalanine (Juth and Munthe, 2011). And so, if successful, a newborn screening programme for PKU would reduce morbidity (or, in some cases, mortality) in the population.

However, there are two related reasons that, when taken together, generate the conclusion that *Public Goods Account*_Outcome_ cannot account for why screening programme for PKU should be classed as an instance of a public health activity. The first, most obvious reason arises from the fact that PKU belongs to the category of *rare—*indeed, *very* rare (Juth and Munthe, 2011)—diseases. On the standard understanding, to say that a condition belongs to the category of rare diseases is to say that it affects no more than 5 in 10,000 people (European Commission, 2024). Rare diseases, then, *by definition* have small patient populations (Magalhaes, 2022). The second, related reason has to do with the idea that the claim that public health activities should produce positive population-level health outcomes is just the claim that they are expected to make a difference by affecting the health of the *many*, in the sense that this difference *should be visible* in aggregate population health figures (Verweij and Dawson, 2007).

In accepting these two reasons, however, we would also be forced to accept that the screening programme for PKU should not be classed as an instance of public health activity. This is because since PKU is (very) rare disease, and since public health activities are expected to make a difference that is visible in aggregate population health figures, screening for PKU in most cases either does not improve the health of the public or, *if it does*, it improves it *non-substantially* and *imperceptibly*. To illustrate this point further, consider the example of implementing a screening programme for PKU in a country like Croatia. Since there are around 32,000 newborns per year in Croatia (Croatian Bureau of Statistics, 2025), and since PKU affects only 1 in (around) 8000 newborns (Petković Ramadža *et al*., 2013), a newborn screening programme for PKU would prevent around *three* cases of PKU in Croatia per year. However, this would seem to offer strong support for the claim that a newborn screening programme for PKU would produce positive population-level health outcomes that have a *non*-substantial and *imperceptible* effect on aggregate population health figures. Insofar as this is true, then, on *Public Goods Account*_Outcome_ a newborn screening programme for PKU would not constitute a public health activity. This, however, seems implausible and something that very few would be ready to accept.

Finally, a proponent of *Public Goods Account*_Outcome_ might, perhaps, respond that activities targeting rare diseases are not problematic for *Public Goods Account*_Outcome_ simply because they do not (or, perhaps, should not) constitute a public health activity in the first place. This seems to be Anomaly’s view, presented in a modified version of the *Public Goods Account*:

Public health should only be concerned with the provision of health-related public goods for which there is a significant demand, or *would be* significant demand if potential consumers of the good had the accurate information about the likely costs and benefits (both moral and monetary) of providing the relevant public good (2011, p. 256, *emphasis in original*).

Accepting this claim leads to the following conclusion. Since PKU is a rare disease that affects *very few people*, there would presumably be no (or is no) *significant demand* for the screening programme aimed at detecting and selecting for treatment those at risk of the condition. Therefore, we have a compelling reason to exclude a screening programme for PKU from the list of public health activities.

While noteworthy, this response is ultimately unsuccessful. The most salient problem with Anomaly’s proposed modification is that ‘demand’ for a health-related public good simply does not seem to be a plausible condition for classing something as a public health activity. Standardly, the claim that there is demand for something is just the claim that there are enough potential consumers who have a desire for that good and who are willing to pay the price for it. But in accepting this claim, we would also be forced to seriously consider the idea that a state should provide a public good, such as, say, national defence, only if there are enough people who desire it and are willing to pay for it. This, however, is something that virtually no one would accept. A state should provide national defence *regardless* of whether there is a sufficient number of people who desire it.

We can explain this in terms of *interests*, rather than desires. On this view, in some cases, there will be an interest for some goods to obtain *even though* the goods in question might not be strictly *desired* by *any* individual (Dawson, 2011). Much the same is true of ‘convergent interests’, a ‘more strongly public’ category of interests that may be expressed in terms of individual interests and that can only be fulfilled by the provision of public goods (Dawson, 2011). On this view, then, it is in our convergent interest that the public good of national defence is obtained, should the unlikely event of an invasion from a foreign country occur, *despite* the fact that there might be individuals who do not strictly desire it or are not willing to pay for it. If this is correct, then we would also have sufficiently compelling reasons to accept the idea that the availability of a screening programme for *PKU* might be in our ‘convergent interest’, despite the fact that many of us would not desire it or be willing to pay for it. That is, like with other public goods, it is in our convergent interest that there is an available screening programme that can, *at least in principle*, benefit *everyone*, in terms of preventing a rare instance of PKU. Therefore, insofar as this is correct, the objection that *Public Goods Account*_Outcome_ cannot account for public health activities that are aimed at addressing (very) rare diseases remains unrefuted.

## Conclusions

In this paper, I examined the *Public Goods Account*, according to which public health activities should be concerned with the provision of health-related public goods, and argued that it is inadequate. Understood as *Public Goods Account*_Medicine_, it cannot account for a number of public health activities that produce public goods by using obviously *non*-medical means and those that are not *exclusively* medical. Understood as *Public Goods Account*_Outcome_, it cannot account for genuine public health activities that produce positive population-level effects, but that are not public goods *tout court* or *pure* public goods. Furthermore, *Public Goods Account*_Outcome_ is problematic because there are public health activities that produce outcomes that do constitute public goods, but these are *not* health-related in the sense that they *do not* produce positive population-level effects on people’s health, or if they do, they do so imperceptibly, which does not warrant the status of a public health activity.

## Notes

1 See for example: Giubilini (2025), Reznek (2022), Richman (2004), and Tengland (2015) who highlight the importance of concepts such as ‘global health’, ‘health’ and ‘enhancement’ respectively.

2 I borrow this from (Boonin, 2008). Admittedly, his claim is about the importance of defining ‘punishment’. However, I think that the same line of reasoning applies to other activities and practices more broadly, including public health activities.

3 I borrow this general idea of ‘specification’ from Richardson (2000).

4 I thank an anonymous reviewer for raising this point.

5 I am grateful to an anonymous reviewer for this point.

6 Here, I understand loneliness and sexual loneliness as a mix of strongly subjective feelings that one’s desired quantity or quality of social and sexual connections is unfulfilled (Hawkley and Cacioppo, 2010).
